# Characterization and Temperature Dependence of Arctic *Micromonas polaris* Viruses

**DOI:** 10.3390/v9060134

**Published:** 2017-06-02

**Authors:** Douwe S. Maat, Tristan Biggs, Claire Evans, Judith D. L. van Bleijswijk, Nicole N. van der Wel, Bas E. Dutilh, Corina P. D. Brussaard

**Affiliations:** 1 Department of Marine Microbiology and Biogeochemistry, NIOZ Royal Netherlands Institute for Sea Research, and University of Utrecht, P.O. Box 59, 1790 AB Den Burg, Texel, The Netherlands; douwe.maat@nioz.nl (D.S.M.); tristan.biggs@nioz.nl (T.B.); clevans@noc.ac.uk (C.E.); judith.van.bleijswijk@nioz.nl (J.D.L.v.B.); 2 Ocean Biogeochemistry & Ecosystems Research Group, National Oceanography Centre, Southampton, European Way, Southampton SO14 3ZH, UK; 3 Electron Microscopy Center Amsterdam, Department of Cell Biology and Histology, Academic Medical Center, University of Amsterdam, Meibergdreef 15, 1105 AZ Amsterdam, The Netherlands; n.n.vanderwel@amc.uva.nl; 4 Theoretical Biology and Bioinformatics, Utrecht University, 3584 CH Utrecht, The Netherlands; bedutilh@gmail.com; 5 Centre for Molecular and Biomolecular Informatics, Radboud University Medical Centre, 6525 GA Nijmegen, The Netherlands

**Keywords:** Arctic algal viruses, climate change, infectivity, *Micromonas* virus, prasinovirus, temperature, virus-host interactions

## Abstract

Global climate change-induced warming of the Artic seas is predicted to shift the phytoplankton community towards dominance of smaller-sized species due to global warming. Yet, little is known about their viral mortality agents despite the ecological importance of viruses regulating phytoplankton host dynamics and diversity. Here we report the isolation and basic characterization of four prasinoviruses infectious to the common Arctic picophytoplankter *Micromonas*. We furthermore assessed how temperature influenced viral infectivity and production. Phylogenetic analysis indicated that the putative double-stranded DNA (dsDNA) *Micromonas polaris* viruses (MpoVs) are prasinoviruses (Phycodnaviridae) of approximately 120 nm in particle size. One MpoV showed intrinsic differences to the other three viruses, i.e., larger genome size (205 ± 2 vs. 191 ± 3 Kb), broader host range, and longer latent period (39 vs. 18 h). Temperature increase shortened the latent periods (up to 50%), increased the burst size (up to 40%), and affected viral infectivity. However, the variability in response to temperature was high for the different viruses and host strains assessed, likely affecting the Arctic picoeukaryote community structure both in the short term (seasonal cycles) and long term (global warming).

## 1. Introduction

Marine phycovirology, i.e., the study of viruses infecting marine eukaryotic algae, started with the lytic viruses infectious to the picophytoplankter *Micromonas pusilla* [1,2,3,4,5]. The genus *Micromonas* (class Mamiellophyceae) is ubiquitous, occurring from tropical to polar regions, and is readily infected by viruses [3,6,7,8,9]. The majority of *Micromonas* virus isolates belong to the double-stranded DNA (dsDNA) prasinoviruses [3,4,5,9], although a dsRNA *Micromonas* virus has also been reported [10,11]. The prasinoviruses are considered the most abundant group of marine phycodnaviruses [12] and virus abundances show synchrony with their hosts’ temporal dynamics consistent with infection [13,14].

*Micromonas* is a globally important prasinophyte, which typically dominates the picophytoplankton fraction in marine Arctic waters [15,16,17,18,19,20,21,22]. Previous studies have shown that Arctic *Micromonas* forms a separate ecotype from lower latitude strains [16,21] adapted to grow at temperatures between 0 and 12 °C (with an optimum around 6–8 °C [16]). Considering Arctic sea surface temperature over the year to be in the range of −1 to a maximum 7 °C [23,24,25] and steadily increasing as a result of global warming (0.03–0.05 °C per year over the 21st century [24]), the *Micromonas* polar ecotype species (tentatively named *M. polaris*; [26]) can be expected to belong to the picophytoplankton predicted to benefit from a warming Arctic region [24,27,28,29]. Despite this predicted increase in abundance and relative share of picophytoplankton in the changing Arctic Ocean, it is still unclear how the viruses infecting the picophytoplankton are affected by changes in temperature. Little is known about Arctic phycoviruses in general, and to our knowledge, no viruses infectious to Arctic *Micromonas* species have yet been brought into culture [30,31,32].

Changes in an environmental variable, such as temperature, may directly affect virus infectivity and/or more indirectly impact virus proliferation due to alterations in the metabolic activity of the host [33]. Thus far the thermal stability of psychrophilic marine virus-host interactions has only been assessed for several phage-bacterium systems [34,35], despite the potential for special physiological adaptations by cold-adapted hosts and viruses [36,37]. It is likely that different viruses infecting the same host strain have distinct responses to shifting environmental factors and therefore environmental change may drive virus selection and host population dynamics. Nagasaki and Yamaguchi [38] found that the temperature ranges for successful infection were different for two virus strains infecting the raphidophyte *Heterosigma akashiwo* and that the host strain sensitivity to infection varied according to the temperature. Furthermore, temperature regulates growth by controlling cellular metabolic activity [39], which has been proportionally related to latent period length and burst sizes for *Vibrio natriegens* phages [40]. Recently, Demeroy and colleagues [41] demonstrated that temperature-regulated growth rates of *Micromonas* strains that originated from the English Channel were responsible for shortened latent periods and increased viral burst sizes upon infection. Ongoing change in the Arctic necessitates a better understanding of how Arctic phycoviruses are affected by temperature.

Here we report on the isolation of four *Micromonas* viruses from the Arctic. In addition to determining their viral characteristics (capsid morphology and size, genome type and size, latent period, phylogeny, host range, burst size, virion inactivation upon chloroform and freezing treatment), we investigated the impact of temperature change on virus infectivity and production. We hypothesize that (i) viral infectivity will increase with temperature, and (ii) increasing temperatures will stimulate virus production (shorter latent periods and higher burst sizes). For testing the latter hypothesis, we performed one-step virus growth experiments at a range of temperatures representative of the extremes over the polar growth season (0.5–7 °C) [23].

## 2. Materials and Methods

### 2.1. Isolation and Culturing

The *Micromonas* host TX-01 was isolated from Kongsfjorden, Spitsbergen, Norway (78°55.073′ N, 12°24.646′ E) on the 19 April 2014, by making an end-point, 10-fold dilution series of fjord water in F/4 medium (based on Whatman glass microfiber GF/F filtered, autoclaved fjord water; [42]). The other *Micromonas* species and strains used were obtained from the Bigelow National Center for Marine Algae and Microbiota (culture collection of marine phytoplankton (CCMP) coded strains; West Boothbay Harbor, ME, USA), the Culture Collection Marine Research Center of Göteborg University (LAC38; Göteborg, Sweden), and the Roscoff Culture Collection (RCC coded strains; Roscoff, France).

*Micromonas* TX-01 was classified based on its position in a Maximum-Likelihood dendrogram (Supplement Figure S1) of 18S rRNA sequences (1574 valid columns) of *Micromonas* strains with clade designations A–E after Slapeta et al. [43] and Ea after Lovejoy et al. [16]. Analysis was done using Randomized Axelerated Maximum Likelihood (RAxML) [44] implemented in the ARB software package [45]. *Micromonas* TX-01 (1051 Bp) was added to the tree using ARB Parsimony. Neighbor-Joining analysis gave a similar tree topology, whereby the tree was rooted using *Mantoniella squamata*. Primers 328F and 329R were used to amplify a part of the small subunit (SSU) ribosomal RNA gene of the *Micromonas* host according to Romari and Vaulot [46]. The same primers plus internal primer 528F were used for sequencing. Isolate TX-01 clustered in clade Ea which is composed of only Arctic clones [16]. Recently it has become clear that the genus *Micromonas* is not made up by solely *M. pusilla,* but instead consists of distinct genetic lineages and new species are described [6,26,47]. The strains in the Ea cluster are recently described as a new species of *Micromonas*, i.e., *M. polaris* [26], and with pending approval we consider TX-01 to be a putative *M. polaris* strain.

*Micromonas* species and strains (Table 1) were cultured in Mix-TX medium, a 1:1 mixture of f/2 medium [42] and artificial seawater [48] enriched with Tris-HCl and Na_2_SeO_3_ [3], under a light:dark cycle of 16:8 h. Light was supplied by 18W/965 OSRAM daylight spectrum fluorescent tubes (München, Germany) at intensities of 70–90 μmol quanta m^−2^ s^−1^. Cultivation temperatures for the different *Micromonas* species and strains used for testing the host range of the virus isolates are listed in Table 1.

The standard temperature at which the *M. polaris* strains used for the virus infection experiments were cultured was 3 °C. For investigating the effect of temperature on the viral growth cycle and virus infectivity, the host strains had been acclimated to various other temperatures (0.5, 2.5, 3.5, and 7 °C for TX-01; and 7 °C for RCC2257 and RCC2258) for several months prior to experimentation. Although the host strain LAC38 is not, the TX-01 and the RCC strains are obligate low-temperature strains, as they did not grow at 15 °C.

Four virus strains were isolated from the waters around Spitsbergen and were named MpoV as they infect Arctic *M. polaris* [26]. MpoV-44T was isolated during winter in 2006 using *Micromonas commoda* strain LAC38 (formerly known as *M. pusilla* [47]), and MpoV-45T to 47T during spring and summer in 2014 and 2015, respectively, using *M. polaris* TX-01 (Table 2). The reason that a low-temperature acclimated LAC38 culture was used for the isolation of MpoV-44T was due to the lack of available Arctic *Micromonas* host strains at the time of isolation. The lytic virus isolate MpoV-44T was isolated by adding whole seawater (15% *v/v*) to an exponentially growing culture of *M. commoda* LAC38 (acclimated to grow at 3 °C), and MpoV-45T, 46T, and 47T by adding 25% *v/v* 0.2 µm filtered (polyethersulfone membrane filter; Sartopore Midicap, Sartorius A.G. Goettingen, Germany) seawater to exponentially growing *M. polaris* TX-01 (standard culturing at 3 °C, but isolation was performed at 4 °C). MpoV-46T was the only one isolated from Storfjorden; the others came from Kongsfjorden (Table 2). Clearing of the infected algal cultures as compared to the non-infected control cultures was indicative of lysis. The lytic agents were confirmed as biological as the obtained lysates could be successfully propagated when 0.2 µm filtered, but not when autoclaved. The lysates were made clonal by end-point dilution (10-fold dilutions) and were maintained by regularly infecting exponentially growing host cultures with 10% *v/v* earlier produced lysates.

### 2.2. Virus Growth Characteristics

To obtain (comparative) information about the latent period and viral burst size of the four MpoVs isolated, viral growth experiments were performed in triplicate at 3 °C in 100 mL Erlenmeyer flasks with an exponentially growing algal host culture of TX-01 and freshly made 0.2 µm filtered (polyethersulfone membrane filter; Sartopore Midicap, Sartorius A.G. Goettingen, Germany) viral lysates. Culture conditions were as described above for host culturing. The virus to host ratio was 10–60:1 (on average 25 ± 12), at all times sufficient to allow one-step viral growth curves. The host strain TX-01 was chosen as a model host system because it was indigenous to this Arctic region and isolated together with three of the four new MpoVs. Growth medium equal to the volume of the lysate was added to non-infected control cultures (in triplicate). Host cell and viral abundances were sampled every 6–24 h post infection (p.i.) at in situ temperatures. Flow cytometry was used to enumerate algae in unpreserved samples, which were kept chilled until analysis whereas samples for virus enumeration were fixed immediately after sampling.

Algal samples were counted using a Becton Dickinson (Becton Dickinson, Franklin lakes, NJ, USA) FACSCalibur benchtop flow cytometer (equipped with a 488 nm argon laser), with the trigger set on red chlorophyll autofluorescence [49]. Viral abundances were determined on fixed samples (final concentration 0.5% glutaraldehyde, EM-grade; Sigma-Aldrich, St. Louis, MO, USA) that were snap-frozen in liquid nitrogen and stored at −80 °C until analysis. Thawed virus samples were diluted in TE buffer (10 mM Tris-Base, 1 mM EDTA, pH 8.0), stained with the nucleic acid-specific green fluorescent dye SYBR Green-I (Invitrogen, Thermo Fisher, Waltham, MA, USA) and analyzed according to Brussaard [50]. *Micromonas* virus clusters were discriminated by a higher green fluorescence (similar to [50], and [9]).

### 2.3. Host Range

A range of *Micromonas* species and *M. polaris* strains were tested for susceptibility to infection by the four MpoVs (Table 1). Five hundred microliters of viral lysate was added to 4.5 mL of exponentially growing host, after which the lysis of the culture was monitored by visual inspection (clearing of the culture compared to non-infected control cultures). Cultures which had not lysed after 3 weeks were considered resistant to the lytic Arctic MpoVs. Lysed cultures were screened for virus production using flow cytometry.

### 2.4. Ultrastructure Analysis by Transmission Electron Microscopy (TEM)

For ultrastructural analysis by TEM, thin sectioned samples were prepared. Briefly, exponentially growing algal cells of *M. polaris* RCC2258 were infected with the respective virus, after which samples (3–6 tubes of 15 mL per sampling point) were taken at several time points within the latent period. Samples were prefixed with glutaraldehyde (EM-grade; 0.5% final concentration) for 30 min on ice. Algal cells were concentrated by low-speed centrifugation (3200× *g*, 10 min, 4 °C), after which the supernatant was decanted and the pellets were transferred to 1.5 mL Eppendorf tubes (three per tube) using a Pasteur pipet. These samples were further concentrated by centrifugation (3200× *g*, 10 min), followed by the transfer of two of the pellets into one Eppendorf tube and another round of centrifugation (3200× *g*, 10 min). Finally, these samples were fixed with glutaraldehyde (EM-grade; 2% final concentration) in 1 mL citrate-phosphate buffer (0.1 M Na_2_HPO_4_·12 H_2_O, 9.7 mM citric acid, pH 7.2) containing 2.5 mM CaCl_2_ on ice.

After fixation, the algae were washed in distilled water, osmicated for 60 min in 1% OsO_4_ in water, and washed again in distilled water with centrifugation steps in between to spin down the algae. After the last spin down, the supernatant was removed and the algae were re-suspended in the remaining volume. An equal volume of 12% gelatin was added to the algal sample and centrifuged again to a non-compact pellet. The gelatin was solidified on ice and after 20 min a fixative (2% glutaraldehyde) was added to let the gelatin fixate overnight. The gelatin containing the algae was cut into small blocks of 1–2 mm^2^ and dehydrated through a series of ethanols (70%, 80%, 90%, 96%). As a last dehydration step, propylene oxide was used before the samples were embedded in LX-112 resin. After polymerization at 60 °C, ultrathin sections of 90 nm were cut on a Reichert EM UC6 with a diamond knife, collected on Formvar coated grids and stained with uranyl acetate and lead citrate. Sections were examined with a FEI Tecnai-12 G2 Spirit Biotwin electron microscope (Fei, Eindhoven, The Netherlands), and images were taken with a Veleta camera using Radius software (EMSIS, Münster, Germany). The data analysis program within Radius was used to perform measurements of the virus particle size. The capsid diameter was measured for 100–400 virus particles per infection, discriminating intracellular and extracellular particles.

### 2.5. Sensitivity to Chloroform

Recently, a new group of *Micromonas* viruses has been reported to possess a lipid membrane [9]. To test for the presence of a viral lipid membrane in these Arctic strains, fresh viral lysates were exposed to chloroform. This organic solvent is an effective indicator of inner- and outer-viral lipid membranes [51,52]. Aliquots of 1 mL were incubated in 10% and 50% (*v/v*) chloroform for 10 min, after which the chloroform was separated by centrifugation (4000× *g*, 5 min) and the aqueous phase containing the viruses was recovered (in new 1.5 mL Eppendorf tubes). Tubes were left overnight at 3 °C to allow any remaining chloroform to evaporate. Treated lysates were added to exponentially growing cultures in 5 mL borosilicate tubes (10% *v/v* final concentration; total volume 5 mL) and incubated at standard light conditions and 3 °C. Non-infected negative controls received the same volume of media. Tubes were screened for lysis twice a week for three weeks. 

### 2.6. Genome Size

MpoV lysates (~25 mL) were partially purified from cell debris and bacteria by centrifugation at 10,000× *g* for 30 min at 4 °C using a fixed angle rotor (type F 34-6-38) with conical adapters to fit the 30 mL Nalgene Oak Ridge centrifuge tubes in a Eppendorf 5810R centrifuge (Hamburg, Germany). Viral genome sizes were determined by Pulse Field Gel Electrophoresis (PFGE) according to Baudoux and Brussaard [53]. In short, the clarified supernatant was decanted and viral particles were concentrated by ultracentrifugation (184,000× *g* for 2 h at 8 °C, using a fixed-angle rotor Beckman Coulter type 50.2Ti, in a Beckman Coulter Optima XPN-80 ultracentrifuge) (Pasadena, CA, USA). Pellets were resuspended in 150 μL SM buffer (0.1 M NaCl, 8 mM MgSO_4_·7 H_2_O, 50 mM Tris-HCl, 0.0005% (*w/v*) glycerin), after which agarose plugs were prepared by mixing equal volumes of molten 1.5% (*w/v*) agarose (InCert; Lonza Group Ltd., Basel, Switzerland) with the virus concentrate in plastic molds. Plugs were incubated overnight at 30 °C in lysis buffer with proteinase K, followed by washing in TE buffer (10:1, pH 8.0) and storage in TE buffer (20:50, pH 8.0) at 4 °C until analysis. Plugged samples were loaded onto 1% SeaKem GTG agarose gels (InCert; Lonza Group Ltd., Basel, Switzerland) prepared in 1 × TBE gel buffer (90 mM Tris-Borate and 1 mM EDTA, pH 8.0) and run in a PFGE Bio-Rad CHEF DR-II cell unit (Bio-Rad, Hercules, CA, USA), and corresponding CHEF DR-II chiller system, filled with 2 L 0.5 × TBE buffer (45 mM Tris-Borate and 0.5 mM EDTA, pH 8.0), pre-cooled at 15 °C. Plugs were loaded with 0.5 × TBE buffer (45 mM Tris-Borate and 0.5 mM EDTA, pH 8.0), at 6 V cm^−1^ with pulse ramps of 20 to 45 s at 14 °C for 22 h. Molecular size markers were included: DNA Lambda ladder plugs (Bio-Rad) and *Saccharomyces cerevisiae* DNA ladder plugs (Bio-Rad). Gels were visualized in a FluorS imager (Bio-Rad Instrument) after staining with SYBR Green I (1 × 10^4^ of commercial solution, Invitrogen). Viral genome sizes were estimated in comparison to a molecular size marker (*n* ≥ 2). Mean ± standard deviation were determined and the differences were tested by ANOVA (significance level *p* = 0.05) and Holm-Šidák multiple comparisons.

### 2.7. Virus Phylogeny

To determine the phylogenetic relationship between our new Arctic virus isolates and other *Micromonas* viruses, we amplified a part of the DNA polymerase B gene (*polB*) using the primer pair AVS1/AVS2 [54]. The viral lysate was diluted 1:5 in ultrapure water and sonicated (MSE Soniprep 150, London, UK) at an amplitude of 8 µm for 3 × 10 s with intervals of 30 s cooling on ice. Ten microliters of sonicated viral lysate was used as a template in a 50 µL PCR reaction containing 4.0 U of BiothermPlus DNA Polymerase (GeneCraft, Lüdinghausen, Germany), 1 × buffer (including 1.5 mM MgCl_2_), 0.25 mM of each dNTP, 0.8 µM of each primer, and 0.4 mg/mL BSA. Negative controls contained all reagents except the template. PCR cycling included an initial denaturation at 94 °C (4 min) followed by 37 cycles of denaturation at 94 °C (30 s), annealing at 45 °C (30 s), and extension at 72 °C (1 min), followed by a final extension at 72 °C (7 min). Sequencing was performed by BaseClear Ltd. (Leiden, The Netherlands). Based on 178 amino acid positions, a Maximum-Likelihood dendrogram was constructed with RAxML [44] implemented in ARB software [45]. The tree was rooted using *polB* sequences of *Bathycoccus* viruses (HM004432, FJ267515, FJ267518, KF501013, MEHZ011588827). These, and the *polB* sequences of *Ostreococcus* viruses (FJ267496, FJ267500, FJ267508, JN225873) were grouped to obtain a more compact tree. For comparison we added published *polB* sequences of the *Micromonas* virus isolates and contigs of an Arctic metagenome [22] that showed exact overlap with the *polB* fragment that we analyzed. Contig-95-10186 and contig-79-31207 were shorter (127 and 96 amino acid positions, respectively) and were later added to the dendrogram via ARB Parsimony.

### 2.8. Thermal Stability

We studied the effect of different temperatures on virus growth characteristics as well as on virus stability (loss of infectivity). To determine how the virus-host interaction might be affected over a range of different ecologically relevant temperatures, we used our model system of TX-01 with MpoV-45T, as both the host and virus were isolated in the same location and same period. Temperature sensitivity of the viral latent period and burst size were examined by one-step viral growth experiments at a range of growth temperatures, i.e., 0.5, 2.5, 3.5, and 7 °C. This range of temperatures represents natural water temperatures during the Arctic growth season [23,24,25]. Additionally, to test whether there are any species- and/or strain-specific responses to temperature, we furthermore tested both MpoV-44T and 45T on RCC 2257 and RCC2258 at 3 and 7 °C, whereby 3 °C represents the spring sea surface temperature around Spitsbergen (origin of TX-01) and southern Beaufort Sea (origin of RCC2257 and RCC2258; [55] and 7 °C represents the maximum Arctic summer temperatures (e.g., [23,56]). MpoV-44T and 45T were chosen as representative virus model systems because of their different viral growth characteristics with both being isolated from Kongsfjorden. Algal host and virus samples were taken regularly (every 6–8 h in the first 24 h and every 12–24 h for the rest of the experiment) and analyzed by flow cytometry as described above [49,50]. Other culture conditions were the same as for the virus growth experiments described above.

Viral infectivity was determined after exposure at −196, −80, −20, 0, 3, 7, and 15 °C and determined using the Most Probable Number (MPN) assay on the host strain RCC2258. This host strain was used instead of TX-01, because the latter did not grow well in the 5 mL tubes that we used for the assays (the large amount of dilutions and replicates did not allow the use of larger tubes or flasks). Aliquots of virus lysates (3 mL) were exposed to the different temperatures for 24 h when the exposure temperature was below zero and for 1 h when exposure temperature was above zero. Following exposure, viral lysates were added to the algal host (*n* = 5; 12 × 10-fold dilutions). In each MPN rack one additional row of tubes containing non-infected culture (also 5 mL per tube) served as a negative control. The MPN cultures were incubated at 3 °C under standard light conditions and were inspected at least once a week for 3 weeks for lysis. The titers were determined with the MPN Assay Analyzer [57] and data were normalized to the highest value, i.e., 9.8 × 10^8^, 4.6 × 10^9^, 2.1 × 10^1^, and 4.6 × 10^9^ mL^−1^ for MpoV-44T, 45T, 46T, and 47T, respectively. Statistics were carried out in SigmaPlot 13.0 (Systat Software Inc., Chicago, Il, USA). Differences between the viruses and temperature treatments were tested by ANOVA (*n* = 3, significance level *p* = 0.05) and Holm-Šidák multiple comparisons, either directly or after log transformation.

### 2.9. Diversity and Abundance in Metagenomes

To assess the diversity of MpoV in diverse marine environments, we searched the contigs generated by the Tara Oceans consortium [58], as well as KEGG Environmental sequences for MpoV homologs using blastn [59]. All hits had an E-value < 10^−30^. Hit regions were excised from the contigs, aligned with the four MpoV sequences using Clustal Omega 1.2.0 [60], and converted into a phylogenetic tree using PhyML 3.0.1 [61] with the HKY85 model of substitution; four discrete gamma categories; shape parameter: 1.074; invariant proportion: 0.455; and transition/transversion ratio: 4.540. Finally, we assessed the ubiquity and abundance of all the MpoV-related sequences in the Tara Oceans samples [58]. Abundance was determined by mapping 2.5 billion metagenomic sequencing reads from 26 Tara Oceans metagenomes to the contig fragments using Burrows-Wheeler Aligner (BWA-MEM algorithm) with default parameters [62], with the number of mapped reads reflecting the relative abundance in the original samples. The IDs of the 26 screened metagenomes were: ERR594313.1, ERR598949.1, ERR598972.1, ERR598982.1, ERR599023.1, ERR599039.1, ERR599095.1, ERR594320.1, ERR598950.1, ERR598976.1, ERR598994.1, ERR599025.1, ERR599053.1, ERR599122.1, ERR594324.1, ERR598962.1, ERR598977.1, ERR599001.1, ERR599027.1, ERR599068.1, ERR594325.1, ERR598966.1, ERR598979.1, ERR599007.1, ERR599035.1, and ERR599078.1.

## 3. Results

### 3.1. Basic Virus Characteristics

**Figure 1:**
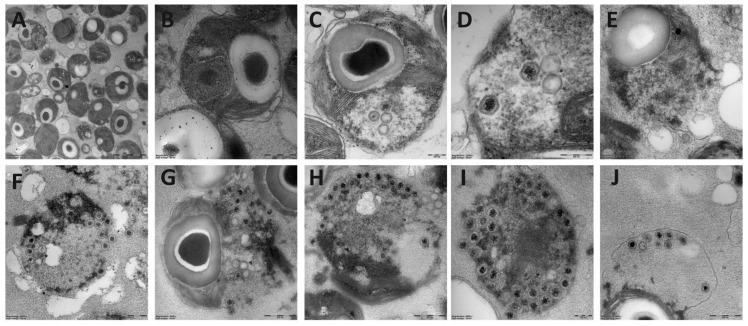
Transmission electron micrographs of thin sections of the uninfected *Micromonas* strain TX-01 (**A**,**B**), and infected with virus MpoV-44T (**C**,**D**), 45T (**E**), 46T (**F**–**H**), and 47T (**I**,**J**). Scale bar represents 200 nm (**A**,**C**,**D**,**I**) or 500 nm (**B**,**E**–**H**,**J**).

The transmission electron microscope analysis of the four Arctic *Micromonas* viruses revealed virus-like particles in the cytoplasm of the host with a hexagonal shape (icosahedral symmetry) and a thick outer layer surrounding an electron-dense inner core (Figure 1). We detected no significant difference between the diameter of the different isolates nor for the intra- versus extracellular virus particles. The diameters of the virus particles were 119 ± 8, 121 ± 14, 120 ± 12, and 119 ± 9 nm respectively for MpoV-44T, 45T, 46T, and 47T. Furthermore, chloroform treatment revealed that all viruses lost their infectivity upon treatment with chloroform, indicative for the presence of a lipid membrane. 

**Figure 2:**
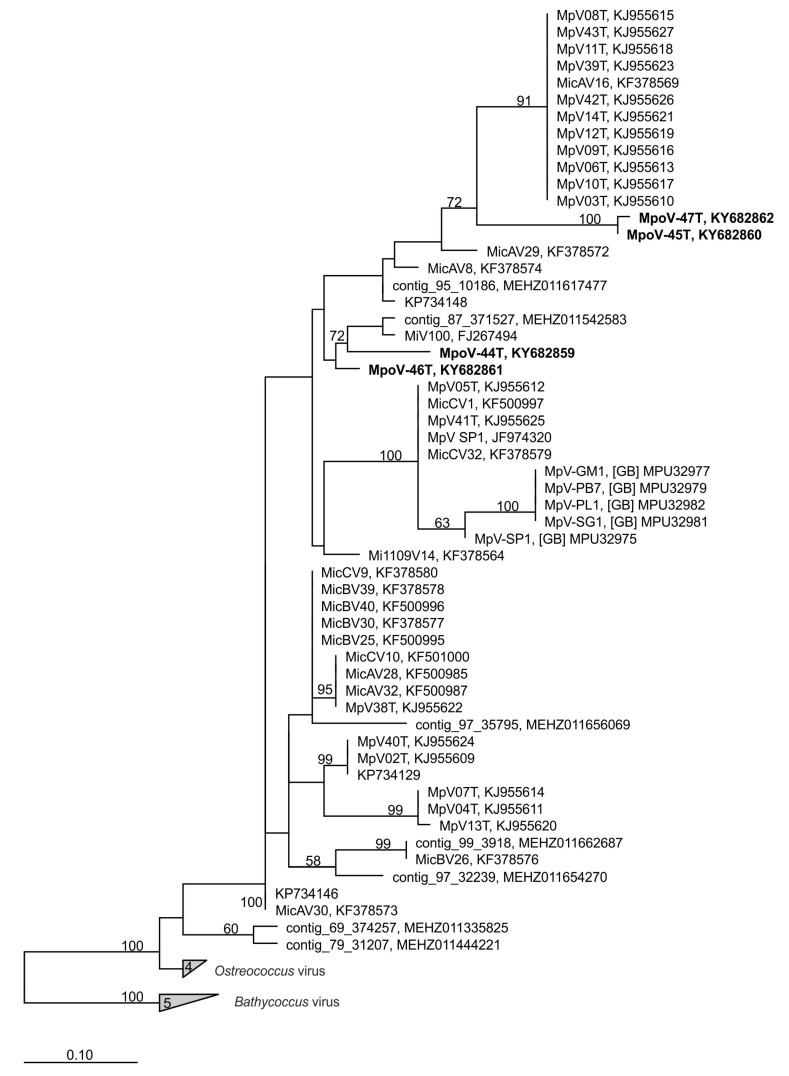
Position of the four *Micromonas polaris* viruses (MpoVs) (in bold) in a maximum likelihood dendrogram (100 bootstrap replicates), based on a multiple alignment of 178 amino acid positions of DNA polymerase B (*polB*). Only nodes with bootstrap values >50% are displayed. Virus strains and accession numbers are indicated. “Contigs” are *polB* sequences extracted from an Arctic marine metagenome [22]. The tree was rooted using *polB* sequences of *Bathycoccus* viruses.

Cytograms of the viruses stained with the nucleic acid-specific dye SYBR Green I showed high green fluorescence signatures, similar to other known dsDNA *Micromonas* viruses (Supplement Figure S2; [9,50]). The dsDNA nature of the MpoV genomes was confirmed by the positive results from the PCR amplification of the partial DNA polymerase B gene (*polB*) using AVS1/AVS2 primers that were originally designed for dsDNA prasinoviruses [54]. The *polB* phylogeny based on inferred amino acid sequences indeed grouped the newly isolated MpoVs with other *Micromonas* viruses, but did not show a close match (i.e., >13 amino acid substitutions) to any of the other Arctic sequences (Figure 2). MpoV-45T and MpoV-47T were highly similar to each other (1 amino acid difference), but clearly distinct from MpoV-46T and 44T (>31 amino acids difference). MpoV-44T differed from MpoV-46T in 17 amino acid positions. The viral genome sizes of MpoV-45T, 46T, and 47T, (191 ± 3 Kb estimated by PFGE) were not significantly different from each other (one-way ANOVA *p* > 0.818, *n* = 4, 2, and 2, respectively) but were significantly smaller (*p* < 0.008) than the genome of MpoV-44T which displayed a genome size of 205 ± 2 Kb (*n* = 3; Table 2, Supplement Figure S4).

The virus isolates were specific for the genus *Micromonas* (Supplement Table S1), but were not species-specific. For example, MpoV-44T was isolated on *M. commoda* LAC38, but also infected TX-01, *M. pusilla*, and *M. polaris* strains (Roscoff Culture Collection; Table 1). Besides the host strain TX-01, RCC2257 and 2258 were also sensitive to infection by all four MpoVs. MpoV-44T displayed the broadest host range and was the only virus that could infect *Micromonas* strains LAC38, CCMP1545, RCC461, and 834 that grow at higher temperatures (8, 15, or 20 °C). No relationship could be established between virus infectivity or host susceptibility to infection based on the time of isolation, geographical origin, or host culture temperature.

**Figure 3:**
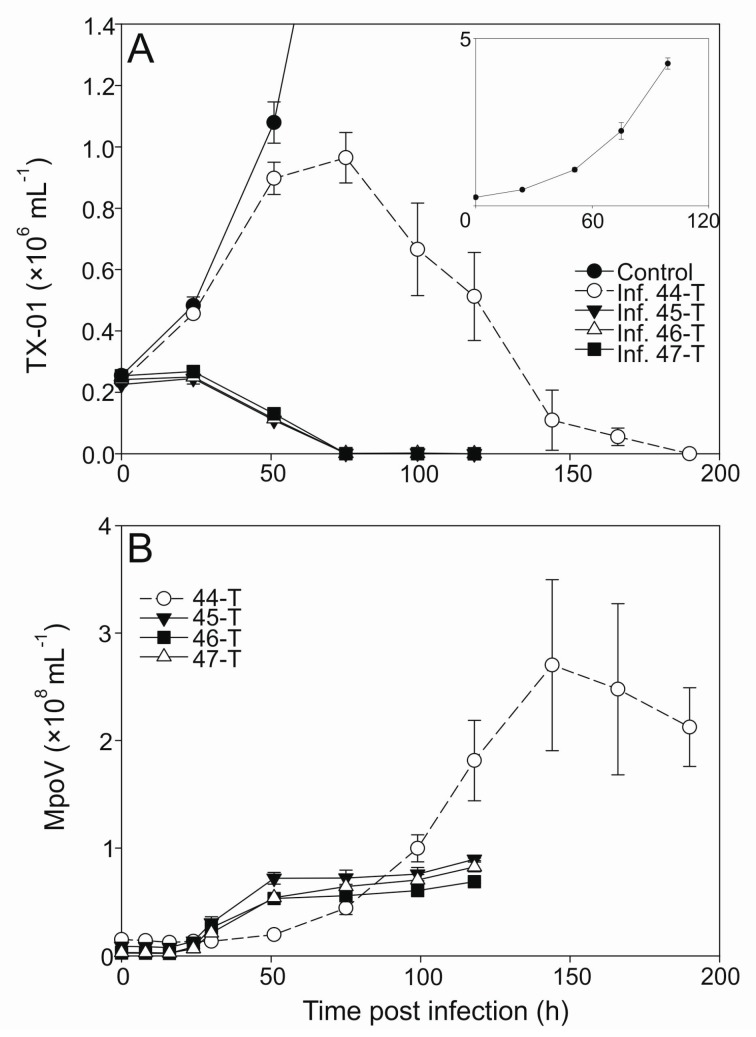
Abundances of *Micromonas* cells (×10^6^ mL^−1^) and viruses MpoV-44T, 45T, 46T, and 47T (×10^8^ mL^−1^) infecting host strain TX-01. Panel (**A**) shows the algal abundances (mean ± standard deviation (S.D.) over time, with the filled circles depicting the non-infected control cultures, open circles depicting the cultures infected with MpoV-44T, filled triangles depicting the ones infected with MpoV-45T, closed triangles depicting the ones infected with MpoV-46T, and the filled squares depicting the ones infected with MpoV-47T. The inlay panel shows the growth of the non-infected controls in detail. Panel (**B**) shows the viral abundances (mean ± S.D.) over time, with the symbols corresponding to panel (**A**), i.e., each virus is depicted by the same symbol as the culture it infected.

One-step infection was observed for all the MpoV lytic virus growth cycles except for MpoV-44T when propagated on TX-01 (Figure 3A). TX-01 kept growing for the two days following virus addition to a higher extent than commonly observed [63,64]. However, this was not observed for other host strains, e.g., RCC2257, RCC2258, and LAC38 (Supplement Figure S3). MpoV45T, 46T, and 47T showed similar infection dynamics and latent periods, i.e., 16–24 h with a median of 18 h (Figure 3B; Table 2). The median latent period for MpoV-44T on host strain TX-01 was, at 39 h, twice as long. Viral burst sizes did not differ significantly for the four MpoVs on TX-01 (one-way ANOVA; *p* = 0.317) and the averages varied between 233 and 296 viruses produced per lysed host cell (Table 2).

**Figure 4:**
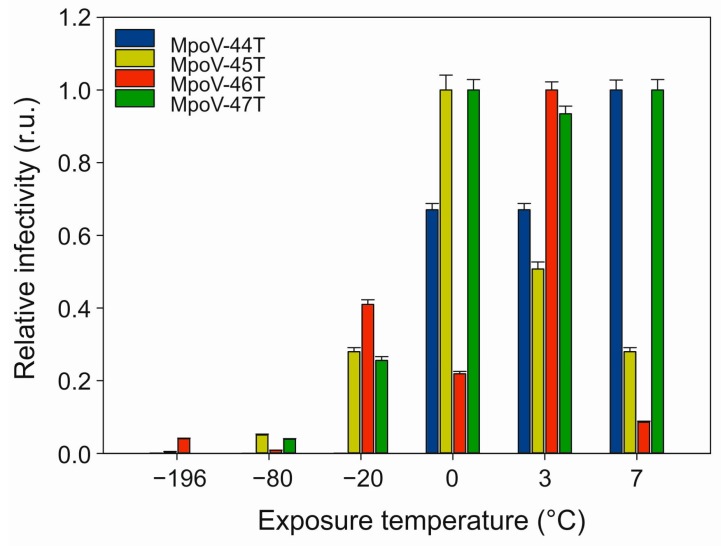
Effects of temperature exposure on the infectivity of MpoV-44T, 45T, 46T, and 47T (actual infection assay performed at 3 °C). The *x*-axis depicts the exposure temperature and the *y*-axis depicts the relative infectivity (normalized to highest infectivity) of the virus as determined by the most probable number (MPN) dilution assay. r.u. stands for relative units. Error bars show standard error (*n* = 5).

Testing the sensitivity of the four Arctic MpoVs to temperature showed that all viruses lost most of their infectivity after 24 h exposure to temperatures < −20 °C (Figure 4). After a −20 °C treatment, only 1% of MpoV-44T remained infective while the other viruses retained over 25% of their infectivity when compared to the treatment at 3 °C. Moreover, the variability between the viruses in response to non-freezing temperatures was high. MpoV-45T and 46T displayed relatively narrow tolerance ranges, with maximal infectivity after the 0 °C and 3 °C treatments, respectively. MpoV-44T and 47T retained infectivity at the highest temperature tested (7 °C), with MpoV-47T being the least sensitive to temperature (consistent infectivity at 0–7 °C).

### 3.2. Temperature Dependent Virus Production

**Figure 5:**
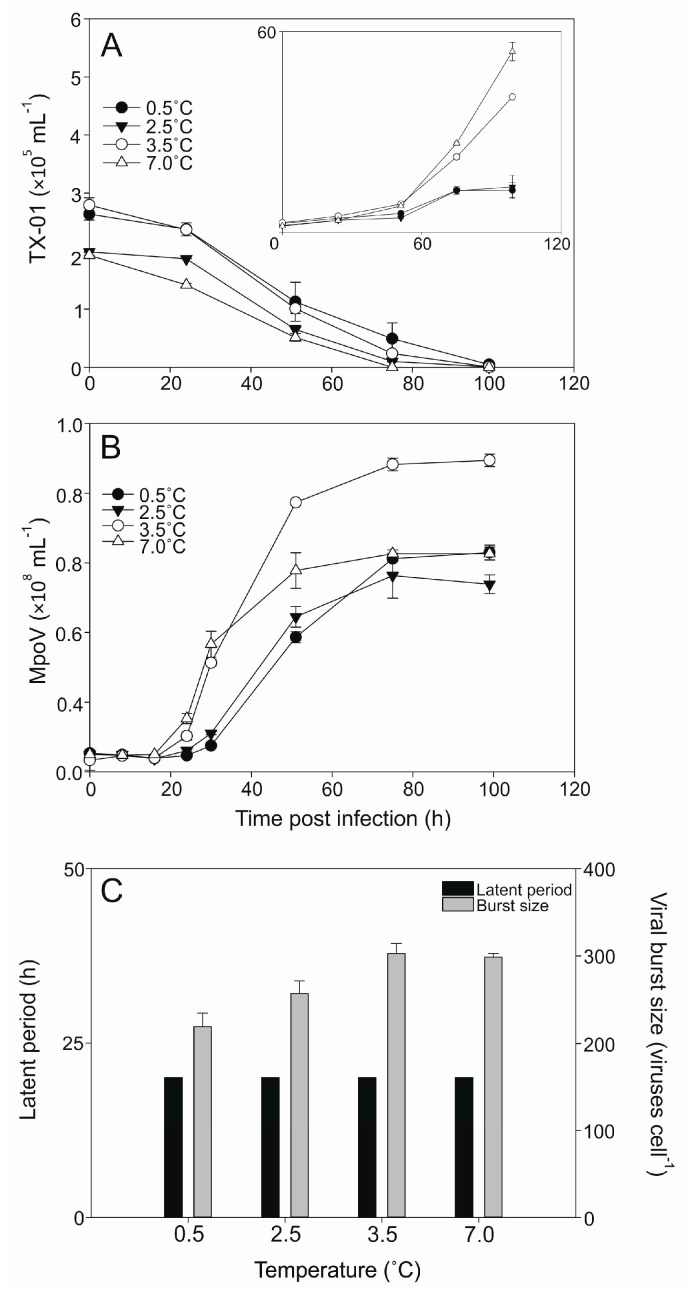
Abundances of *Micromonas* strain TX-01 (×10^5^ mL^−1^) and virus MpoV-45T **(**×10^6^ mL^−1^**)** tested at 0.5, 2.5, 3.5, and 7.0 °C. Panel (**A**) shows the algal abundances (mean ± S.D.) over time, with filled circles representing 0.5 °C, filled triangles representing 2.5 °C, open circles representing 3.5 °C, and open triangles representing 7.0 °C. The inlay panel shows the growth of the non-infected controls. Panel (**B**) shows the viral abundances (mean ± S.D.) over time, with the symbols corresponding to panel A, i.e., each virus is depicted by the same symbol as the culture it infected. Panel (**C**) depicts the median viral latent periods (black bars; determined with an 8 h sampling resolution) and viral burst sizes (grey bars; mean ± S.D.).

The lysis dynamics of the MpoV-45T infecting host TX-01 was similar for all four temperatures tested (0.5, 2.5, 3.5, 7 °C; Figure 5A) despite increasing exponential growth rates of the host (0.40 ± 0.05, 0.49 ± 0.06, 0.66 ± 0.01, 0.85 ± 0.02 d^−1^, respectively). The latent period of MpoV-45T did not change with temperature (16–24 h; Figure 5B), but the viral burst sizes did show significant differences between 0.5, 2.5, and 3.5 °C (one-way ANOVA; 0.001 < *p* < 0.019) and declined with lower temperatures by 15% and 28% for 2.5 and 0.5 °C, respectively, compared to 3.5 °C (Figure 5C). 

**Figure 6:**
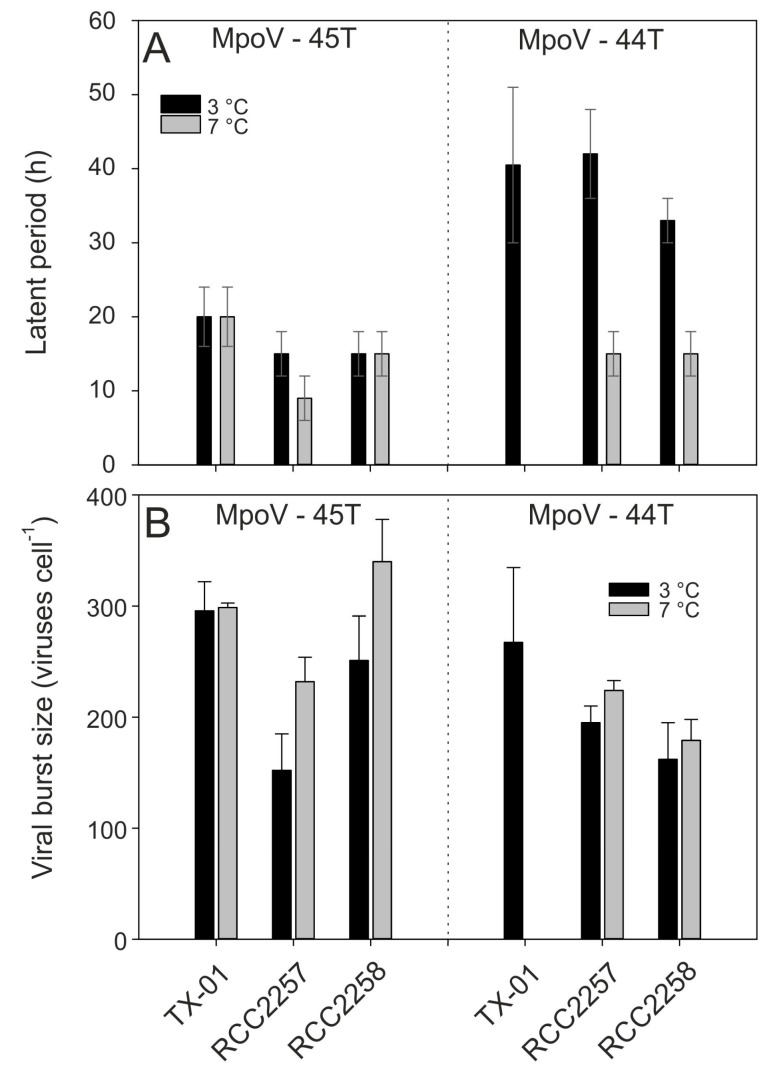
Median latent periods (**A**) and mean burst sizes (**B**) of MpoV-45T (left panels) and MpoV-44T (right panels) infecting host TX-01, RCC2257, and RCC2258 at 3 °C (black bars) and 7 °C (grey bars). Note that the TX-01 data are from the same as in Figure 5. The range bars in panel A depict the actual time interval on which the latent period is based. The error bars in panel B depict the standard deviation (S.D.). Statistical analysis of inter- and intra-strain differences are depicted in Supplement Table S2.

Assessing the other virus-host combinations for temperature sensitivity (7 °C compared to 3 °C), the virus growth characteristics revealed host-specific effects. MpoV-45T showed a shorter latent period at higher temperature when RCC2257 was the host (from 12–18 h to 6–12 h), whereas no such effects were observed on hosts TX-01 and RCC2258; Figure 6A). Moreover, while TX-01 (infected with MpoV45T) did not show an increase in burst size from 3.5 to 7.0 °C (but did from 2.5 to 7.0 °C; see above), RCC2257 and RCC2258 showed increased burst sizes at 7 °C by respectively 150% and 140% (one-way ANOVAs; *p* < 0.045; Figure 6B, Supplement Table S2). 

There were also virus-specific responses to temperature, as an increase from 3 °C to 7 °C showed a stronger effect on the latent periods of MpoV-44T rather than that of MpoV-45T, reducing it by roughly 50% from >30 h to 12–18 h on both hosts. Moreover, the viral burst sizes of MpoV-44T showed only a significant increase at 7 °C on host RCC2257 (one-way ANOVA; *p* = 0.044), which was also smaller than that for MpoV-45T (115% compared to 150%). Irrespective of temperature, on host RCC2258 the viral burst sizes of MpoV-45T were higher than those of MpoV-44T, but for host RCC2257 no such difference was observed (two-way ANOVA; two- *p* < 0.001 and *p* < 0.916, respectively).

### 3.3. Diversity and Abundance in Metagenomes

**Figure 7:**
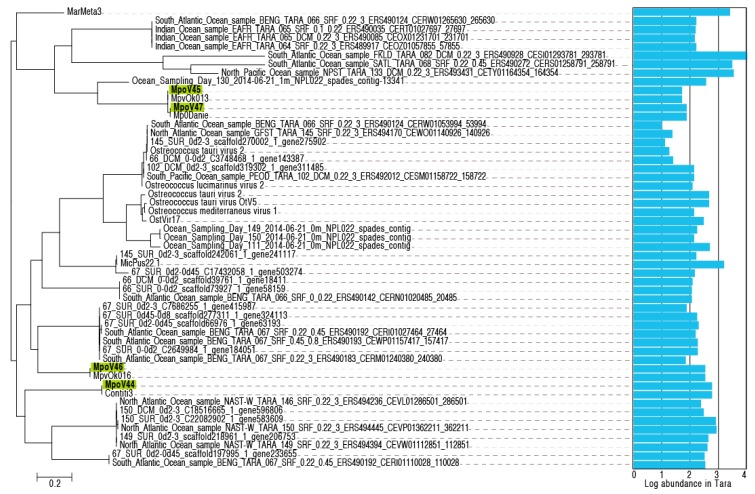
Unrooted maximum likelihood phylogeny of MpoV-related sequences from various studies, and their abundance in environmental metagenomes. Abundance is expressed as the total number of aligned reads out of 2.5 billion reads in 26 Tara Oceans datasets.

Finally, we assessed the diversity and abundance in the metagenomes by screening sequence databases for homologs of the amplified nucleotide region. The phylogenetic tree in Figure 7 shows that MpoVs are part of a family of viruses whose sequences were previously detected in marine samples from around the world from various studies including Tara Oceans [58] and the Ocean Sampling Day.

## 4. Discussion

To our knowledge this is the first report of the isolation and characterization of phycoviruses from polar marine waters. Similar to other reports of *Micromonas* virus isolates, and consistent with the *Phycodnaviridae*, the virus-like particles accumulated in the cytoplasm of the host cells [9]. The particles of the four MpoV were morphologically similar (no significant variance in virus particle size, i.e., on average 120 nm), and all contained a lipid membrane (sensitive to chloroform). Lipid-containing MpoVs were first reported by Martínez Martínez and colleagues [9]. These authors were able to clearly and convincingly divide nineteen newly isolated *Micromonas* viruses, across an area spanning the North Sea to the Mediterranean Sea, into two groups based on (i) their sensitivity to infection of LAC38 or CCMP1545, (ii) genome size (206 ± 6 Kb (*n* = 12) vs. 191 ± 4 Kb (*n* = 8)), and (iii) presence of a lipid membrane. Strikingly, all LAC38-infecting viruses with larger genomes contained a lipid membrane, whereas the smaller genome sized CCMP1545-infecting ones did not. Our data show a similar larger genome size for MpoV-44T which infects LAC38 (205 ± 2 Kb, in contrast to the 191 ± 3 Kb genomes of the other MpoVs), but in our case all MpoVs contained a lipid membrane. 

Molecular phylogeny inferred from the amino acid sequences of the DNA polymerase gene B fragments established that the four Arctic MpoV isolates grouped distinctly with the other dsDNA *Micromonas* viruses belonging to the genus Prasinovirus. The genus Prasinovirus infects *Ostreococccus* and *Micromonas* species and is one of the six virus genera belonging to the *Phycodnaviridae* family; eukaryotic algal viruses with large dsDNA genomes (100–560 Kbp) [65,66]. A recent metagenomic survey (Tara Ocean Expedition) revealed that prasinoviruses are the most abundant group of phycodnaviruses in the oceans [12]. The newly isolated MpoVs did not group together; instead MpoV-44T and 46T were phylogenetically distinct, both from each other and from MpoV-45T and 47T. Furthermore, it is clear from the phylogenic analysis based on *polB* that the Arctic *Micromonas* viruses do not form a separate cluster. Screening the Tara Oceans’ contigs and the KEGG Environmental database for homologs of the amplified nucleotide region of our MpoV isolates revealed a worldwide distribution and high diversity on thermal stability (i.e., from the Greenland Sea to the temperate regions to Antarctica and in waters from −1.6 to 17.3 °C, Table S3). These results confirm that the *Micromonas* viruses and their relatives are globally dispersed, show a high degree of genotypic diversity, and are ecologically relevant.

There was no general relationship between the phylogenies of the virus and host strains as revealed by Martínez Martínez et al. [9] for viruses infecting temperate *Micromonas* strains, but MpoV-44T could be distinguished from the other Arctic MpoV isolates based on its capability to virally infect *M. commoda* LAC38. Although LAC38 was being cultured at low temperature (3 °C) at the time of virus isolation, it is originally a temperate *Micromonoas* strain (Baltic Sea) [67]. We cannot exclude that the isolation of MpoV-44T on a different host is underlying its intrinsic differences to the other MpoVs isolated 8–9 years later using a local Arctic *Micromonas* host strain. These differences may also be due to MpoV-44T having been isolated during midwinter and years before the other MpoVs (isolated in summertime during two consecutive years). Successional patterns for marine virus communities with associations to temperature and host dynamics have been demonstrated (e.g., [68]). Even though Arctic *Micromonas* still grows well at low temperatures (0.4 d^−1^ at 0.5 °C, this study) and low light (0.2 d^−1^, [16]), photosynthesis may not be possible during part of the Arctic winter. Several *Micromonas* species however exhibit phagotrophy (e.g., *M. polaris* CCMP2099; [69]) that could serve as an alternative energy source to maintain growth and/or virus production during the winter (see also [22]). Yet, our study shows that MpoV-44T is well adapted to relatively fast and high production of infective progeny at relatively higher temperatures, which makes it more likely that advection of relatively warm Atlantic water from the West Spitsbergen Current (WSC) [70] was responsible for being able to isolate MpoV-44T in winter. Water temperature in autumn of the year of isolation was in fact higher than the average of the preceding years [71]. Additionally, the ability of MpoV-44T to successfully infect *Micromonas* strains growing at higher temperatures up to 20 °C seems indicative that this specific virus has a high temperature tolerance. Still, a relatively high temperature optimum for a virus occurring in a cold environment could theoretically be an adaptation to be less virulent in order to avoid extinction of the host [14,72]. The relatively long latent periods and reduced infectivity of MpoV-44T at low temperatures would effectuate such low virulence for the slow growing hosts during the winter months. Then in the following more productive season, when the host growth rates increase, the latent period of MpoV-44T shortens and burst sizes increase to be able to keep in sync with host growth. Intriguingly, MpoV-47T also displayed thermostability, with an infectivity optimum at 7 °C. MpoV-47T was isolated only 2 months later than the temperature sensitive MpoV-45T (infectivity optimum at 0 °C), indicative of a high degree of diversity of virus thermostability in Arctic waters. 

At temperatures above zero, the infectivity data do not confirm our first hypothesis that MpoV infectivity increases with temperature. The viral response to higher temperatures (0–7 °C) was highly variable and strain-specific. Two of the four virus isolates (MpoV-45 and 46T) even showed a loss of infectivity above 0 °C. All of the virus isolates, except for MpoV-44T, were able to maintain over 25% of their infectivity after being frozen at –20 °C. This suggests that these viruses are well able to withstand the freezing process during ice formation, a property which would maintain high titers during winter. Similar findings have been reported for Arctic marine bacteriophages [73] and dsDNA algal viruses during winter in a seasonally frozen pond [74]. Cottrell and Suttle [5] reported relatively high decay rates for MpV-SP1, however, this virus strain originated from subtropical waters and the decay rates were largely determined by sunlight (UV intensity). There is limited knowledge of dsDNA algal virus thermal stability and the mechanisms underlying the loss of infectivity have not been elucidated [9,41,53,75,76,77]. Only a few cold-active viruses (i.e., viruses that successfully infect hosts at 4 °C or below) have been brought into culture and all are bacteriophages [35,78,79]. Variability in MpoVs’ temperature sensitivity demonstrates a specificity of infection efficiency related to temperature. Hypothesizing that our data are generally applicable, seasonal temperature shifts could regulate *Micromonas* host and virus succession. The infectivity loss at higher temperature (7 °C) for MpoV-45T and MpoV-46T shortens their window of optimal activity during the warmer summer months. Considering that the range of temperatures we tested are ecologically relevant for the Artic seas (water temperatures between –1 and 7 °C; [23]), our results indicate the need to determine the causal processes such as the means of virus entry and conformational changes in the virus particle (e.g., viral capsid proteins and lipid membrane properties).

When propagated on the putative *M. polaris* strain TX-01, MpoV-44T displayed a much longer median latent period than the other Arctic MpoVs (39 vs. 18 h, respectively). A comparably long latent period had, until recently, only been described for the dsRNA virus MpRV infecting *M. commoda* LAC38 (36 h; [10]). However, Baudoux and colleagues [14] reported a latent period of 27–31 h for a dsDNA virus infecting *Micromonas* isolates from the English Channel growing at 20 °C. The latent period of MpoV-44T displayed a strong temperature-dependence, i.e., with a temperature increase of 4 °C, the time of viral release decreased by >15 h (latent period 12–18 h; approximately 50% of the latent period at 3 °C). Although Baudoux et al. [14] did not find a correlation between differences in latent period (or burst size) with the host growth rates for the isolated MicVs, Demory et al. [41] reported for the virus-host model system Mic-B/MicV-B (virus infecting largely Clade B strains) an inverse relationship of the viral latent period with the host growth rate (whereby the growth rates were affected by the host culture temperature). We did not find such a relationship with growth rate for the latent periods of MpoV-44T growing on TX-01, but did find a similar significant linear relationship when infecting host RCC2258 (r^2^ = 0.952, *p* = 0.018). Virus MpoV-45T did not show a dependency on host growth rate (TX-01, RCC2258, and RCC2257), but instead showed a shortened latent period on host RCC2257 at the highest temperature (7 °C compared to 3 °C). Furthermore, the latent period of MpoV-44T was strongly affected by temperature whereas increasing temperature only shortened the MpoV-45T latent period on RCC2257. On host TX-01, the latent period was unaffected over the whole range of 0.5 to 7 °C, however, we found a steeper increase of viruses with increasing temperature, i.e., virus production rates of 0.18, 0.37, 1.4, and 1.6 × 10^5^ viruses h^−1^ between 16 and 30 h for 0.5, 2.5, 3.5, and 7.0 °C, respectively. While the data confirm our second hypothesis that the temperature increase stimulates MpoV production (through shortened latent periods, enhanced production rate and/or higher burst sizes), there is nonetheless a virus-specific response for the range of temperatures tested. Instead we found a high variability in response for the different virus isolates and host strains. When looking at a temperature increase from 3 to 7 °C, most virus-host combinations in our study showed enhanced viral burst sizes with a temperature-regulated increase in the host growth rates (0.53–0.59 d^−1^ at 3 °C and approximately 1.2-fold higher at 7 °C). However, MpoV-45T infecting TX-01 did not show this increase from 3 to 7 °C, but did exhibit an increasing burst size with temperatures from 0.5 up to 3.5 °C (growth rate TX-01 increased from 0.40 to 0.66 d^−1^, respectively). Hence, the optimum temperature for virus production was not the same as the optimum host growth temperature. Wells and Deming [78] showed a similar situation in which phage 9A, infecting the psychrophile *Colwellia psychrerythraea* strain 34H, had a burst size optimum at −1 °C while the host’s growth rate optimum was at 8 °C. These authors suggested that specific virally encoded enzymes have their own optimum temperatures. 

Temperature strongly regulates Arctic *Micromonas* growth rates with increasing growth rates up to 7 °C (this study; [16,29]). These temperatures are at or above the present summer sea surface temperatures in the lower latitude regions of the Arctic [23,25]. At the time that TX-01 was isolated (half April), in situ picophotoeukaryotic gross growth rates were 0.58 d^−1^ at temperatures between 1 and 2 °C (Maat and Brussaard, unpublished data). By the end of May, the temperatures and consequently the growth rates had increased to 2–3 °C and 1.1 d^−1^, respectively. This 20–50% growth rate increase is similar to TX-01 in the present study over the same temperature range. Our results indicate that over an Arctic growing season with increasing temperatures and host growth rates, viral activity can be expected to increase as a result of the decreasing latent periods and increasing burst sizes. Our data imply that temperature could affect host and virus diversity (strain dynamics), as for MpoV-45T with host TX-01 the latent period and viral burst size did not further change above 3 °C, but with other hosts (RCC2257 and 2258) and other viruses (MpoV-44T) the latent periods shortened and/or burst sizes increased to several extents. The different susceptibilities of viral infectivity to temperature, with a tolerance for the highest temperatures for MpoV-44T, strengthened this idea even more. Tarutani et al. [80] showed how within the observed abundances of *Heterosigma akashiwo* and the lytic virus HaV, a successional shift in clonal composition occurred due to differences in susceptibility/resistance of the host to the viruses. Based on our data a similar shift in strain and clonal composition of *Micromonas* and MpoV may occur, not only due to differences in susceptibility to the viruses but also because of differences in virus proliferation success at different temperatures [33]. 

In summary, the present study describes the first isolation and characterization of viruses infecting a cold-adapted polar phytoplankter. The relevance seems high, as it concerns the ubiquitous genus *Micromonas* which belongs to the picophytoplankton fraction and is expected to be favored under future Arctic conditions (due to warming and freshening induced vertical stratification; [24,27,28,29]). The Arctic region is warming to a greater extent than lower latitudinal marine waters [23,24] and current summer sea surface temperatures (August 2016) as high as 5 °C above the 1982–2010 mean [25] have been observed. *Micromonas* growth rates will enhance faster and earlier in the season and our study indicates that viral production will likely do the same. We show variable infection dynamics in response to temperature for the different virus-host strain systems examined, which complicates the assessment of the environmental relevance of each isolate. However, we do like to advocate that virus (and host) isolation, characterization of virus-host dynamics, and responses to changing ecologically relevant environmental factors are fundamentally essential to understanding the role of algal viruses in (Arctic) marine waters. The newly isolated viruses make it possible to comprehensively investigate the interactions of these unique virus-host combinations under climate change relevant environmental variables. Joli et al. [22] showed the importance of Arctic *Micromonas* viruses by metagenome sequencing. It would be interesting to investigate the ecological relevance of the strains tested in our study using molecular approaches. In the natural environment, selective effects of temperature may drive (intra)species diversity, potentially affecting the ability of *Micromonas* to respond to the changing conditions of the vulnerable Arctic. Modeling studies could help to comprehend (and predict) the extent to which the Arctic phytoplankton community would be influenced by changes in infection dynamics associated with temperature changes. 

## Figures and Tables

**Table 1:** Lytic activity of the four *Micromonas polaris* viruses (MpoV) against different *Micromonas* species and strains. Columns show from left to right: host strain code, origin of isolation, *Micromonas* species, culturing temperature, and the MpoV strain names. Grey cells mean that the virus from the column is able to infect and lyse the host from the row.

Host Code	Origin	*Micromonas*	Culture Temp.	Lytic Activity against *Micromonas*			
				MpoV-44T	MpoV-45T	MpoV-46T	MpoV-47T
**TX-01**	KF (2014)	*M. polaris*	3 °C				
**LAC 38**	OFN (1998)	*M. commoda* ^1^	3 °C				
**LAC 38**	OFN (1998)	*M. commoda* ^1^	15 °C				
**CCMP 1545**	EC (1950)	*M. pusilla*	15 °C				
**CCMP 2099**	BB (1998)	*Micromonas* sp.	3 °C				
**RCC 461**	EC (2001)	*M. pusilla*	15 °C				
RCC 834 *	EC (1950)	*M. pusilla*	20 °C				
**RCC 2242**	BzS (2009)	*M. polaris*	3 °C				
**RCC 2246**	BS (2009)	*M. polaris*	3 °C				
**RCC 2257**	BS (2009)	*M. polaris*	3 °C				
**RCC 2258**	BS (2009)	*M. polaris*	3 °C				
**RCC 2306**	BS (2009)	*M. polaris*	3 °C				
**RCC 4298**	GS (2014)	*M. polaris*	3 °C				
**RCC 4778**	GS (2014)	*M. polaris*	3 °C				
**RCC 4779**	GS (2014)	*M. polaris*	3 °C				

BB stands for Baffin Bay, BzS for Barents Sea, BS for Beaufort Sea, EC for English Channel, GS for Greenland Sea, KF for Kongsfjorden Spitsbergen, and OFN for Oslofjord Norway. ^1^ formerly known as *M. pusilla*; [47], * original CCMP1545.

**Table 2:** Overview of the origin and basic characterization of the Arctic double-stranded DNA (dsDNA) virus isolates (MpoV-44T, 45T, 46T, and 47T) infecting *Micromonas*. Isolation coordinates Spitsbergen: Kongsfjorden 78°56′28.55″ N, 12°0′2.50″ E, Storfjorden: 77°37′35.26″ N, 20°46′3.74″ E.

MpoV Strain	Geographical Origin Spitsbergen	Date of Isolation	Host Strain of Isolation	Isolation Temperature (°C)	Genome Size (Kbp)	Lipid Membrane	Latent Period (h) *	Burst Size (Viruses Cell^−1^) *
**44T**	Kongsfjorden	December 2006	LAC38	3	205 ± 2	+	30–51	267 ± 67
**45T**	Kongsfjorden	April 2014	TX-01	4	191 ± 2	+	16–24	296 ± 26
**46T**	Storfjorden	August 2015	TX-01	4	192 ± 3	+	16–24	233 ± 7
**47T**	Kongsfjorden	June 2014	TX-01	4	190 ± 6	+	16–24	256 ± 13

* Tested on *M. polaris* TX-01 at 3 °C.

## Supplementary Materials

The following are available online at www.mdpi.com/1999-4915/9/6/134/s1, Table S1: phytoplankton genera used for MpoV infection assays, Table S2: *p*-values belonging to Figure 6B, Table S3: Top 3 BLASTN hits of the isolates against KEGG environmental metagenomes. Figure S1: Phylogeny *Micromonas polaris* TX-01, Figure S2: Cytograms of MpoVs, Figure S3: Virus growth cycle of MpoV-44T infecting different *Micromonas* host strains, Figure S4: PFGE photograph for genome size estimation.

## References

[1] Mayer J.A., Taylor F.J.R. (1979). A virus which lyses the marine nanoflagellate *Micromonas pusilla*. Nature.

[2] Waters R.E., Chan A.T. (1982). *Micromonas pusilla* virus: The virus growth cycle and associated physiological events within the host cells; host range mutation. J. Gen. Virol..

[3] Cottrell M.T., Suttle C.A. (1991). Wide-spread occurrence and clonal variation in viruses which cause lysis of a cosmopolitan, eukaryotic marine phytoplankter, *Micromonas pusilla*. Mar. Ecol. Prog. Ser..

[4] Cottrell M.T., Suttle C.A. (1995). Dynamics of lytic virus infecting the photosynthetic marine picoflagellate *Micromonas pusilla*. Limnol. Oceanogr..

[5] Cottrell M.T., Suttle C.A. (1995). Genetic Diversity of Algal Viruses Which Lyse the Photosynthetic Picoflagellate *Micromonas pusilla* (Prasinophyceae). Appl. Environ. Microbiol..

[6] Worden A.Z., Lee J.-H., Mock T., Rouzé P., Simmons M.P., Aerts A.L., Allen A.E., Cuvelier M.L., Derelle E., Everett M.V. (2009). Green evolution and dynamic adaptations revealed by genomes of the marine picoeukaryotes *Micromonas*. Science.

[7] Not F., Latasa M., Marie D., Cariou T., Vaulot D., Simon N. (2004). A single species, *Micromonas pusilla* (Prasinophyceae), dominates the eukaryotic picoplankton in the Western English Channel. Appl. Environ. Microbiol..

[8] Foulon E., Not F., Jalabert F., Cariou T., Massana R., Simon N. (2008). Ecological niche partitioning in the picoplanktonic green alga *Micromonas pusilla*: Evidence from environmental surveys using phylogenetic probes. Environ. Microbiol..

[9] Martínez Martínez J., Boere A., Gilg I., van Lent J.W.M., Witte H.J., van Bleijswijk J.D.L., Brussaard C.P.D. (2015). New lipid envelope-containing dsDNA virus isolates infecting *Micromonas pusilla* reveal a separate phylogenetic group. Aquat. Microb. Ecol..

[10] Brussaard C.P.D., Noordeloos A.A.M., Sandaa R.-A., Heldal M., Bratbak G. (2004). Discovery of a dsRNA virus infecting the marine photosynthetic protist *Micromonas pusilla*. Virology.

[11] Attoui H., Jaafar F.M., Belhouchet M., de Micco P., de Lamballerie X., Brussaard C.P.D. (2006). *Micromonas pusilla* reovirus: A new member of the family Reoviridae assigned to a novel proposed genus (Mimoreovirus). J. Gen. Virol..

[12] Hingamp P., Grimsley N., Acinas S.G., Clerissi C., Subirana L., Poulain J., Ferrera I., Sarmento H., Villar E., Lima-Mendez G. (2013). Exploring nucleo-cytoplasmic large DNA viruses in Tara Oceans microbial metagenomes. ISME J..

[13] Zingone A., Sarno D., Forlani G. (1999). Seasonal dynamics in the abundance of *Micromonas pusilla* (Prasinophyceae) and its viruses in the Gulf of Naples (Mediterranean Sea). J. Plankton Res..

[14] Baudoux A.C., Lebredonchel H., Dehmer H., Latimier M., Edern R., Rigaut-Jalabert F., Ge P., Guillou L., Foulon E., Bozec Y. (2015). Interplay between the genetic clades of *Micromonas* and their viruses in the Western English Channel. Environ. Microbiol. Rep..

[15] Not F., Massana R., Latasa M., Marie D., Colson C., Eikrem W., Pedrós-Alió C., Vaulot D., Simon N. (2005). Late summer community composition and abundance of photosynthetic picoeukaryotes in Norwegian and Barents Seas. Limnol. Oceanogr..

[16] Lovejoy C., Vincent W.F., Bonilla S., Roy S., Martineau M.J., Terrado R., Potvin M., Massana R., Pedrós-Alió C. (2007). Distribution, phylogeny, and growth of cold-adapted picoprasinophytes in Arctic Seas. J. Phycol..

[17] Balzano S., Marie D., Gourvil P., Vaulot D. (2012). Composition of the summer photosynthetic pico and nanoplankton communities in the Beaufort Sea assessed by T-RFLP and sequences of the 18S rRNA gene from flow cytometry sorted samples. ISME J..

[18] Kilias E., Wolf C., Nöthig E.M., Peeken I., Metfies K. (2013). Protist distribution in the Western Fram Strait in summer 2010 based on 454-pyrosequencing of 18S rDNA. J. Phycol..

[19] Kilias E.S., Nöthig E.M., Wolf C., Metfies K. (2014). Picoeukaryote plankton composition off west Spitsbergen at the entrance to the Arctic Ocean. J. Euk. Microbiol..

[20] Metfies K., von Appen W.J., Kilias E., Nicolaus A., Nöthig E.M. (2016). Biogeography and photosynthetic biomass of arctic marine pico-eukaroytes during summer of the record sea ice minimum 2012. PLoS ONE.

[21] Balzano S., Gourvil P., Siano R., Chanoine M., Marie D., Lessard S., Sarno D., Vaulot D. (2012). Diversity of cultured photosynthetic flagellates in the northeast Pacific and Arctic Oceans in summer. Biogeosciences.

[22] Joli N., Monier A., Logares R., Lovejoy C. (2017). Seasonal patterns in Arctic prasinophytes and inferred ecology of *Bathycoccus* unveiled in an Arctic winter metagenome. ISME J..

[23] Richter-Menge J., Mathis J., Blunden J., Arndt J.S. (2015). The Arctic. State of the Climate in 2015.

[24] ACIA (2005). Chapter 9: Marine Systems. Arctic Climate Impact Assessment.

[25] Timmermans M.-L. (2016). Sea Surface Temperature. http://www.webcitation.org/6ogrhzGOI.

[26] Simon N., Foulon E., Grulois D., Six C., Latimier M., Desdevises Y., Latimier M., Le Gall F., Tragin M., Houdan A. (2017). Revision of the genus *Micromonas* (Manton et Parke) (Chlorophyta, Mamiellophyceae), of the type species *M. pusilla* (Butcher) Manton & Parke and of the species *M. commoda* (van Baren, Bacry and Worden) and description of two new species based on the genetic and phenotypic characterization of cultured isolates. Protist.

[27] Li W.K., McLaughlin F.A., Lovejoy C., Carmack E.C. (2009). Smallest algae thrive as the Arctic Ocean freshens. Science.

[28] Li W.K., Carmack E.C., McLaughlin F.A., Nelson R.J., Williams W.J. (2013). Space-for-time substitution in predicting the state of picoplankton and nanoplankton in a changing Arctic Ocean. J. Geophys. Res. Oceans.

[29] Coello-Camba A., Agustí S., Vaqué D., Holding J., Arrieta J.M., Wassmann P., Duarte C.M. (2015). Experimental assessment of temperature thresholds for Arctic phytoplankton communities. Estuar. Coast.

[30] Brussaard C.P.D., Noordeloos A.A.M., Witte H., Collenteur M.C.J., Schulz K.G., Ludwig A., Riebesell U. (2013). Arctic microbial community dynamics influenced by elevated CO_2_ levels. Biogeosciences.

[31] Lara E., Arrieta J.M., Garcia-Zarandona I., Boras J.A., Duarte C.M., Agustí S., Wassmann P.F., Vaqué D. (2013). Experimental evaluation of the warming effect on viral, bacterial and protistan communities in two contrasting Arctic systems. Aquat. Microb. Ecol..

[32] Payet J.P., Suttle C.A. (2014). Viral infection of bacteria and phytoplankton in the Arctic Ocean as viewed through the lens of fingerprint analysis. Aquat. Microb. Ecol..

[33] Mojica K.D.A., Brussaard C.P.D. (2014). Factors affecting virus dynamics and microbial host–virus interactions in marine environments. FEMS Microbiol. Ecol..

[34] Olsen R.H. (1967). Isolation and growth of psychrophilic bacteriophage. Appl. Microbiol..

[35] Borriss M., Helmke E., Hanschke R., Schweder T. (2003). Isolation and characterization of marine psychrophilic phage-host systems from Arctic sea ice. Extremophiles.

[36] D’amico S., Collins T., Marx J.C., Feller G., Gerday C. (2006). Psychrophilic microorganisms: Challenges for life. EMBO Rep..

[37] Wells L.E., Margesin R., Schinner F., Marx J.-C., Gerday C. (2008). Cold-active viruses. Psychrophiles: From Biodiversity to Biotechnology.

[38] Nagasaki K., Yamaguchi M. (1998). Effect of temperature on the algicidal activity and the stability of HaV (*Heterosigma akashiwo* virus). Aquat. Microb. Ecol..

[39] Toseland A.D.S.J., Daines S.J., Clark J.R., Kirkham A., Strauss J., Uhlig C., Lenton T.M., Valentin K., Pearson G.A., Moulton V. (2013). The impact of temperature on marine phytoplankton resource allocation and metabolism. Nat. Clim. Chang..

[40] Zachary A. (1978). An ecological study of bacteriophages of *Vibrio natriegens*. Can. J. Microbiol..

[41] Demory D., Arsenieff L., Simon N., Six C., Rigaut-Jalabert F., Marie D., Ge P., Bigeard E., Jacquet S., Sciandra A. (2017). Temperature is a key factor in *Micromonas*–virus interactions. ISME J..

[42] Guillard R.R.L., Ryther J.H. (1962). Studies of marine planktonic diatoms: I. *Cyclotella Nana* Hustedt, and *Detonula Confervacea* (CLEVE) Gran. Can. J. Microbiol..

[43] Šlapeta J., López-García P., Moreira D. (2006). Global dispersal and ancient cryptic species in the smallest marine eukaryotes. Mol. Biol. Evol..

[44] Stamatakis A. (2014). RAxML version 8: A tool for phylogenetic analysis and post-analysis of large phylogenies. Bioinformatics.

[45] Ludwig W., Strunk O., Westram R., Richter L., Meier H., Buchner A., Lai T., Steppi S., Jobb G., Förster W. (2004). ARB: A software environment for sequence data. Nucleic Acids Res..

[46] Romari K., Vaulot D. (2004). Composition and temporal variability of picoeukaryote communities at a coastal site of the English Channel from 18S rDNA sequences. Limnol. Oceanogr..

[47] van Baren M.J., Bachy C., Reistetter E.N., Purvine S.O., Grimwood J., Sudek S., Yu H., Poirier C., Deerinck T.J., Kuo A. (2016). Evidence-based green algal genomics reveals marine diversity and ancestral characteristics of land plants. BMC Genomics.

[48] Harrison P.J., Waters R.E., Taylor F.J.R. (1980). A broad-spectrum artificial seawater medium for coastal and open ocean phytoplankton. J. Phycol..

[49] Marie D., Brussaard C.P.D., Thyrhaug R., Bratbak G., Vaulot D. (1999). Enumeration of marine viruses in culture and natural samples by flow cytometry. Appl. Environ. Microbiol..

[50] Brussaard C.P.D. (2004). Optimization of procedures for counting viruses by flow cytometry. Appl. Environ. Microbiol..

[51] Feldman H.A., Wang S.S. (1961). Sensitivity of various viruses to chloroform. Exp. Biol. Med..

[52] Olsen R.H., Siak J.-S., Gray R.H. (1974). Characteristics of PRD1, a plasmid-dependent broad host range DNA bacteriophage. J. Virol..

[53] Baudoux A.-C., Brussaard C.P.D. (2005). Characterization of different viruses infecting the marine harmful algal bloom species *Phaeocystis globosa*. Virology.

[54] Chen F., Suttle C.A. (1996). Evolutionary relationships among large double-stranded DNA viruses that infect microalgae and other organisms as inferred from DNA polymerase genes. Virology.

[55] Mustapha S.B., Larouche P., Dubois J.-M. (2016). Spatial and temporal variability of sea-surface temperature fronts in the coastal Beaufort Sea. Cont. Shelf Res..

[56] Hop H., Falk-Petersen S., Svendsen H., Kwasniewski S., Pavlov V., Pavlova O., Søreide J.E. (2006). Physical and biological characteristics of the pelagic system across Fram Strait to Kongsfjorden. Prog. Oceanogr..

[57] Passmore R., Hsu J., Liu R.X., Tam E., Cai Y., Su W., Frasca J., Brigden S.M., Comeau A.M., Ortmann A.C. 2000. MPN Assay Analyzer. http://www.webcitation.org/6ogxAqLbE.

[58] Sunagawa S., Coelho L.P., Chaffron S., Kultima J.R., Labadie K., Salazar G., Djahanschiri B., Zeller G., Mende D.R., Alberti A. (2015). Structure and function of the global ocean microbiome. Science.

[59] Altschul S.F., Gish W., Miller W., Myers E.W., Lipman D.J. (1990). Basic local alignment search tool. J. Mol. Biol..

[60] Sievers F., Wilm A., Dineen D., Gibson T.J., Karplus K., Li W., Lopez R., McWilliam H., Remmert M., Söding J. (2011). Fast, scalable generation of high-quality protein multiple sequence alignments using Clustal Omega. Mol. Syst. Biol..

[61] Guindon S., Dufayard J.F., Lefort V., Anisimova M., Hordijk W., Gascuel O. (2010). New algorithms and methods to estimate maximum-likelihood phylogenies: Assessing the performance of PhyML 3.0. Syst. Biol..

[62] Li H., Durbin R. (2009). Fast and accurate short read alignment with Burrows-Wheeler transform. Bioinformatics.

[63] Baudoux A.-C., Brussaard C.P.D. (2008). Influence of irradiance on virus-algal host interactions. J. Phycol..

[64] Maat D.S., de Blok R., Brussaard C.P.D. (2016). Combined phosphorus limitation and light stress prevent viral proliferation in the phytoplankton species *Phaeocystis globosa*, but not in *Micromonas pusilla*. Front. Mar. Sci..

[65] Wilson W.H., van Etten J.L., Allen M.J. (2009). The Phycodnaviridae: The story of how tiny giants rule the world. Curr. Top. Microbiol. Immunol..

[66] Clerissi C., Grimsley N., Ogata H., Hingamp P., Poulain J., Desdevises Y. (2014). Unveiling of the diversity of Prasinoviruses (Phycodnaviridae) in marine samples by using high-throughput sequencing analyses of PCR-amplified DNA polymerase and major capsid protein genes. Appl. Environ. Microbiol..

[67] Sahlsten E. (1998). Seasonal abundance in Skagerrak-Kattegat coastal waters and host specificity of viruses infecting the marine photosynthetic flagellate *Micromonas pusilla*. Aquat. Microb. Ecol..

[68] Pagarete A., Chow C.E., Johannessen T., Fuhrman J.A., Thingstad T.F., Sandaa R.A. (2000). Strong seasonality and interannual recurrence in marine myovirus communities. Appl. Environ. Microbiol..

[69] McKie-Krisberg Z.M., Sanders R.W. (2014). Phagotrophy by the picoeukaryotic green alga Micromonas: Implications for Arctic Oceans. ISME J..

[70] Hop H., Pearson T., Hegseth E.N., Kovacs K.M., Wiencke C., Kwasniewski S., Eiane K., Mehlum F., Gulliksen B., Wlodarska-Kowalczuk M. (2002). The marine ecosystem of Kongsfjorden, Svalbard. Polar Res..

[71] Tverberg V., Nilsen F., Goszczko I., Cottier F., Svendsen H., Gerland S. (2008). The warm winter temperatures of 2006 and 2007 in the Kongsfjorden water masses compared to historical data. 8th Ny-Ålesund (NySMAC) Science Managers Committee.

[72] Suttle C.A. (2007). Marine viruses—Major players in the global ecosystem. Nat. Rev. Microbiol..

[73] Wells L.E., Deming J.W. (2006). Modelled and measured dynamics of viruses in Arctic winter sea-ice brines. Environ. Microbiol..

[74] Long A.M., Short S.M. (2016). Seasonal determinations of algal virus decay rates reveal overwintering in a temperate freshwater pond. ISME J..

[75] Tomaru Y., Katanozaka N., Nishida K., Shirai Y., Tarutani K., Yamaguchi M., Nagasaki K. (2004). Isolation and characterization of two distinct types of HcRNAV, a single-stranded RNA virus infecting the bivalve-killing microalga *Heterocapsa circularisquama*. Aquat. Microbiol. Ecol..

[76] Tomaru Y., Tanabe H., Yamanaka S., Nagasaki K. (2005). Effects of temperature and light on stability of microalgal viruses, HaV, HcV and HcRNAV. Plankton Biol. Ecol..

[77] Nagasaki K., Shirai Y., Tomaru Y., Nishida K., Pietrokovski S. (2005). Algal viruses with distinct intraspecies host specificities include identical intein elements. Appl. Environ. Microbiol..

[78] Wells L.E., Deming J.W. (2006). Characterization of a cold-active bacteriophage on two psychrophilic marine hosts. Aquat. Microb. Ecol..

[79] Luhtanen A.M., Eronen-Rasimus E., Kaartokallio H., Rintala J.M., Autio R., Roine E. (2014). Isolation and characterization of phage–host systems from the Baltic Sea ice. Extremophiles.

[80] Tarutani K., Nagasaki K., Yamaguchi M. (2000). Viral impacts on total abundance and clonal composition of the harmful bloom-forming phytoplankton *Heterosigma akashiwo*. Appl. Environ. Microbiol..

