# Supplementary material for: Characterization and Temperature Dependence of Arctic Micromonas polaris Viruses
Source: Viruses. 2017 Jun 2;9(6):134. doi: 10.3390/v9060134 (PMC5490811; doi:10.3390/v9060134)
Supplement: Supplementary file 1 [file viruses-09-00134-s001.pdf]

Supplementary material:

Table S1: Phytoplankton genera and species from different phyla that were tested for infection by the Arctic *Micromonas* virus isolates MpoV-44T, 45T, 46T and 47T. In none of the cultures occurred lysis. Cultures were maintained at 3 °C (except for *Ostreococcus* which was cultured at 15 °C) in MIX-TX medium at 16:8h light:dark and 70–90 μmol quanta m^{-2} s^{-1} (see for details Materials & Methods). Species depicted with TX* are from the Netherlands Institute for Sea Research (NIOZ) TX culture collection and were isolated from Kongsfjorden at the same time as *Micromonas* TX-01. RUG** is from the culture collection of the University of Groningen (RuG), The Netherlands.

Phytoplankton species	Location of isolation
Phaeocystis sp. CCMP1374	Southern Ocean
Phaeocystis antarctica CCMP1871	Southern Ocean
Phaeocystis Antarctica TX17	Southern Ocean
Imantonia sp. TX*	Kongsfjorden
Chrysochromulina sp. CCMP1215	Bellingshausen Sea
Chlamydomonas sp. CCMP681	Bellingshausen Sea
Pyramimonas sp. RuG**	Southern Ocean
Rhodomonas sp. TX*	Kongsfjorden
Proboscia alata TX22	Southern Ocean
Fragilariopsis kerguelensis TX18	Southern Ocean
Chaetoceros dichaeta TX19	Southern Ocean
Chaetoceros debilis TX20	Southern Ocean
Chaetoceros brevis TX21	Kongsfjorden
Thallassiosira hispida TX*	Kongsfjorden
Porosira glacialis CCMP650	Narragansett Bay
Ostreococcus tauri RCC745	Mediterranean Sea

Table S2: *P*-values of the burst size differences between groups, as tested by 1-way ANOVAs, to demonstrate inter- and intra-strain differences (e.g. for RCC2257 increasing temperature leads to significantly increased burst sizes of both viruses, but for RCC2258 this is only found for MpoV-45T). For each host strain, the effect of the used virus (44T x 45T) or temperature (3 °C x 8 °C) on burst size was tested within each separate treatment. Significant differences are depicted in bold.

	3 °C<br>44T x 45T	8 °C<br>44T x 45T	44T<br>3°C x 8°C	45T<br>3°C x 8°C
TX-01	0.493	-	-	-
RCC2257	0.130	0.855	**0.044**	**0.045**
RCC2258	**0.008**	**<0.001**	0.227	**0.003**

Table S3: Top 3 BLASTN hits of the isolates against KEGG environmental metagenomes with relevant information on sampling location and temperature. OSD: Ocean Sampling Day; TARA: TARA Oceans.

Isolate	Campaign	Region	Year	Temp (°C)	Identity	Sample
MpV46T	OSD	Greenland Sea	2014	−1.6	85%	OSD146_2014-06-21_5m_NPL022
	TARA	Southern Ocean	2001	−0.8	84%	85_DCM_0d2-3
	TARA	North Atlantic	2003	17.3	83%	151_SUR_0d2-3
MpV45T	OSD	Greenland Sea	2014	10.1	83%	OSD130_2014-06-21_1m_NPL022
	OSD	North Atlantic	2014	12.8	88%	OSD152_2014-06-20_1m_NPL022
	TARA	Southern Ocean	2012	7.3	81%	82_SRF_0d2-3
MpV47T	OSD	North Atlantic	2014	12.8	87%	OSD152_2014-06-20_1m_NPL022
	OSD	Greenland Sea	2014	10.1	82%	OSD130_2014-06-21_1m_NPL022
	TARA	South Atlantic	2012	7.3	80%	82_SRF_0d2-3
MpV44	TARA	Southern Ocean	2001	−0.8	80%	85_DCM_0d2-3
	TARA	South Atlantic	2009	12.8	80%	67_SUR_0d2-0d45
	TARA	North Pacific	2010	13.2	79%	133_DCM_0d2-3

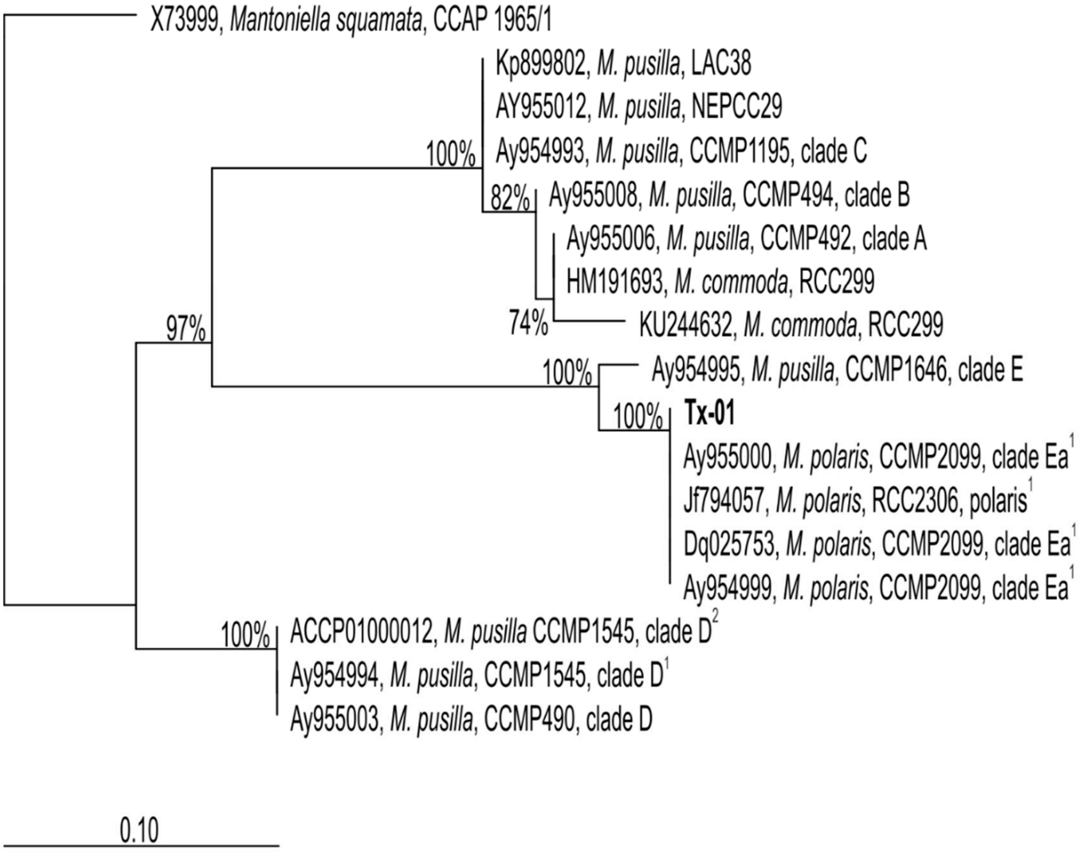

Figure S1: Position of strain TX-01 in a Maximum-Likelyhood dendrogram of 18S rRNA sequences (1574 valid columns) of *Micromonas* strains with clade designations A-E from Slapeta et al. [43] and Ea from Lovejoy et al. [16]. The letter M. stands for the genus *Micromonas*. [1] Simon et al. [48]; [2] Van Baren et al. [47].

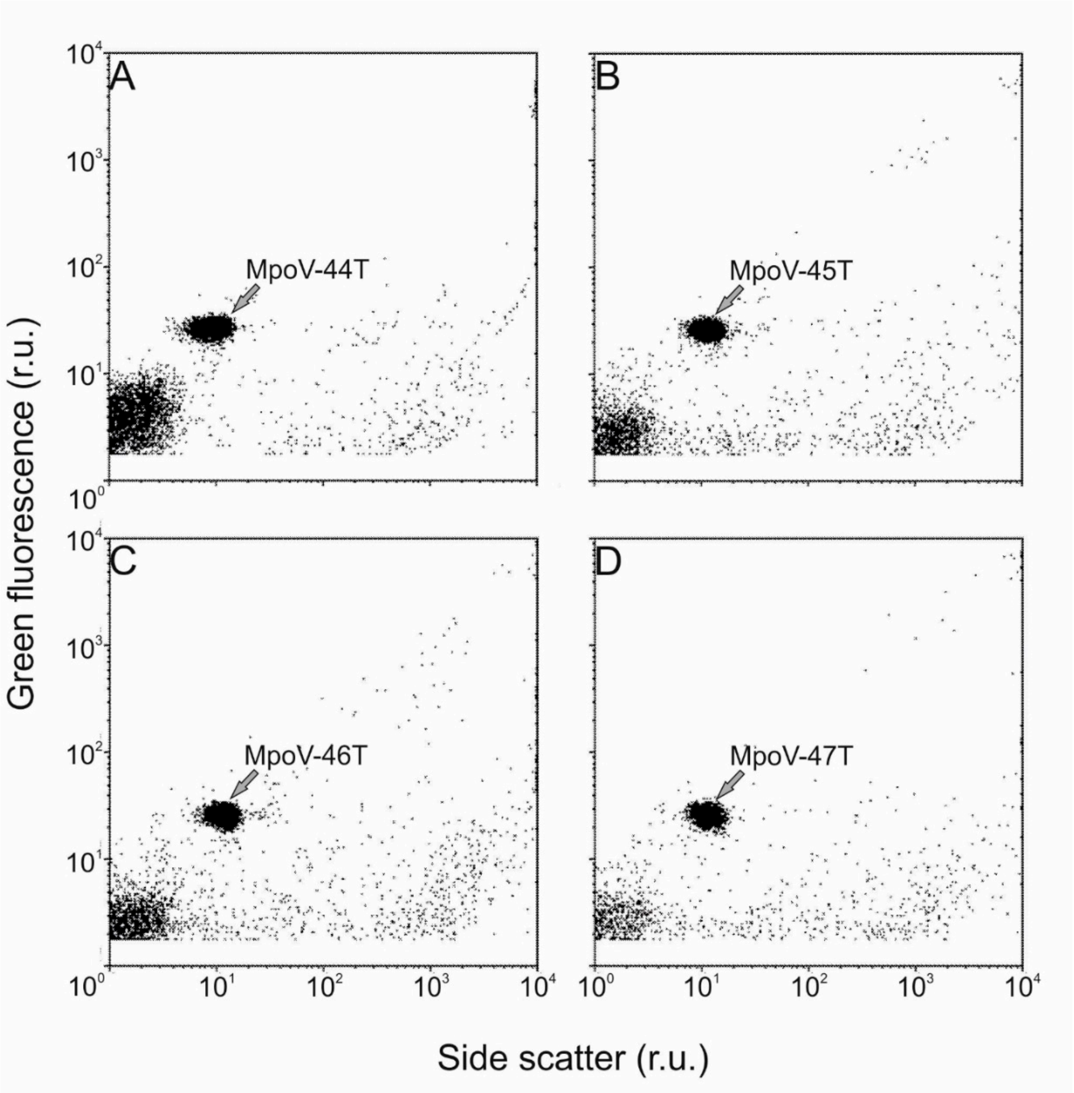

Figure S2: Flow cytograms of the four Micromonas virus strains MpoV-44T, 45T, 46T and 47T (A, B, C, D) with green fluorescence on the Y-axis and side scatter on the X-axis. The grey arrow, which is on the same location in each plot, indicates the MpV cluster.

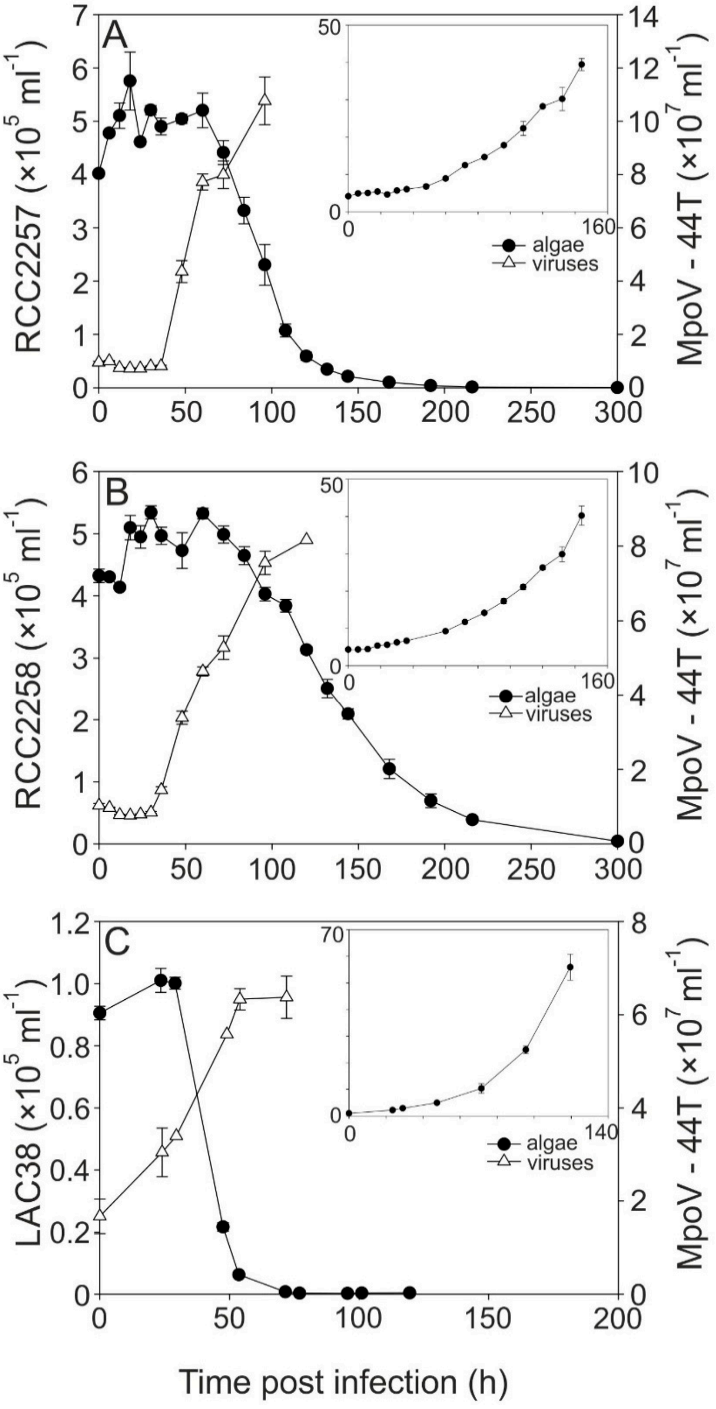

Figure S3: Growth curves of the infection cycle of MpoV-44T infecting M. polaris RCC2257 (A), M. polaris RCC2258 (B) and M. commoda LAC38 (C) as illustration of MpoV-44T - host combinations that do not show the growth extent of the infected TX-01 infected with this virus. Algal cell abundances (mean ± s.d.; n=3) are depicted as filled circles, of which the temporal dynamics of the infected cultures are shown in the main panel and those of the of the non-infected controls in the inlay panel. Viral abundances (mean ± s.d.; n=3) over time are depicted as open triangles. Invisible error bars fall within the symbol.

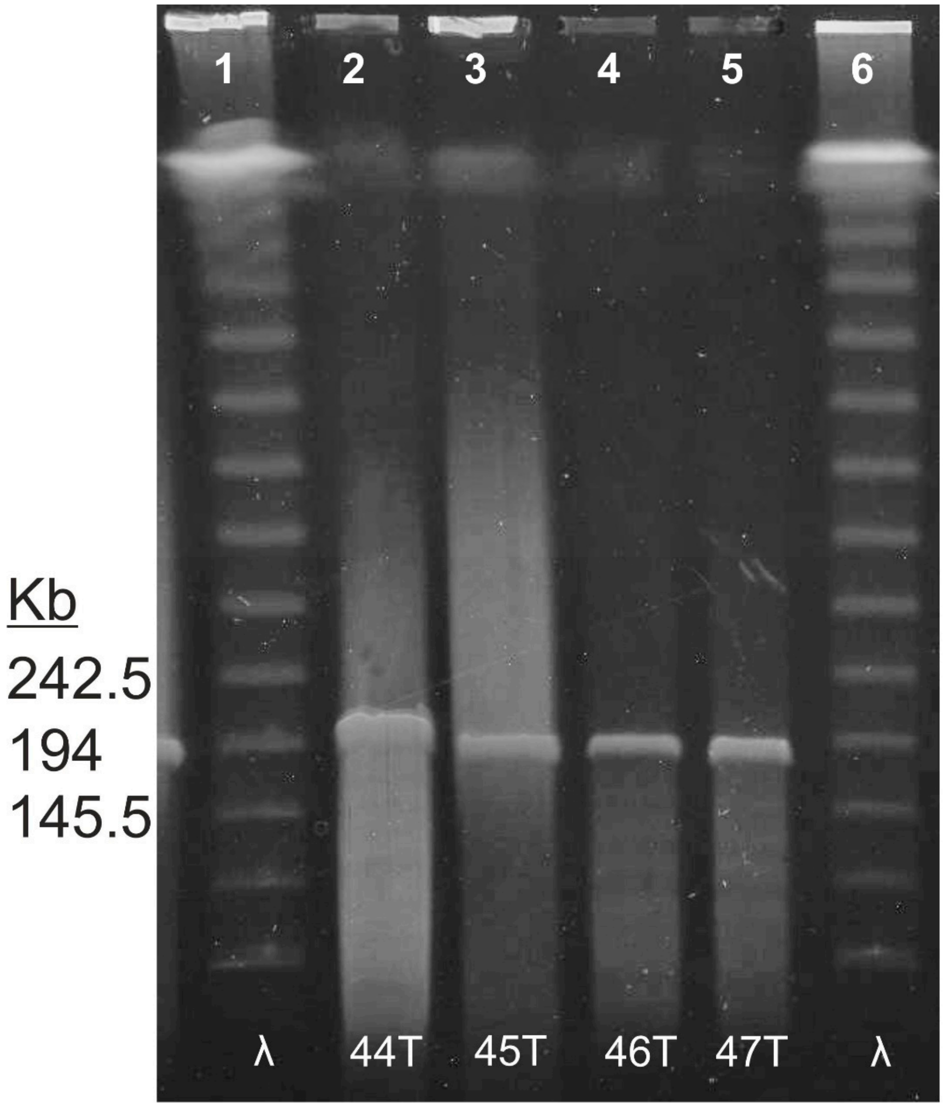

Figure S4: Photograph of a Pulse Field Gel Electrophoresis (PFGE) gel for viral genome size estimation. The virus isolates MpoV-44T to 47T are depicted below lanes 2 to 5. The DNA Lambda ladder is shown in lane 1 and 6 (indicated λ symbol), with the relevant band sizes in Kb on the left.
